# A view of the genetic and proteomic profile of extracellular matrix molecules in aging and stroke

**DOI:** 10.3389/fncel.2023.1296455

**Published:** 2023-11-30

**Authors:** Martina Chmelova, Peter Androvic, Denisa Kirdajova, Jana Tureckova, Jan Kriska, Lukas Valihrach, Miroslava Anderova, Lydia Vargova

**Affiliations:** ^1^Department of Neuroscience, Second Faculty of Medicine, Charles University, Prague, Czechia; ^2^Department of Cellular Neurophysiology, Institute of Experimental Medicine of the Czech Academy of Sciences, Prague, Czechia; ^3^Laboratory of Gene Expression, Institute of Biotechnology of the Czech Academy of Sciences – BIOCEV, Vestec, Czechia

**Keywords:** extracellular matrix, stroke, aging, genes, proteins

## Abstract

**Introduction:**

Modification of the extracellular matrix (ECM) is one of the major processes in the pathology of brain damage following an ischemic stroke. However, our understanding of how age-related ECM alterations may affect stroke pathophysiology and its outcome is still very limited.

**Methods:**

We conducted an ECM-targeted re-analysis of our previously obtained RNA-Seq dataset of aging, ischemic stroke and their interactions in young adult (3-month-old) and aged (18-month-old) mice. The permanent middle cerebral artery occlusion (pMCAo) in rodents was used as a model of ischemic stroke. Altogether 56 genes of interest were chosen for this study.

**Results:**

We identified an increased activation of the genes encoding proteins related to ECM degradation, such as matrix metalloproteinases (MMPs), proteases of a disintegrin and metalloproteinase with the thrombospondin motifs (ADAMTS) family and molecules that regulate their activity, tissue inhibitors of metalloproteinases (TIMPs). Moreover, significant upregulation was also detected in the mRNA of other ECM molecules, such as proteoglycans, syndecans and link proteins. Notably, we identified 8 genes where this upregulation was enhanced in aged mice in comparison with the young ones. Ischemia evoked a significant downregulation in only 6 of our genes of interest, including those encoding proteins associated with the protective function of ECM molecules (e.g., brevican, Hapln4, Sparcl1); downregulation in brevican was more prominent in aged mice. The study was expanded by proteome analysis, where we observed an ischemia-induced overexpression in three proteins, which are associated with neuroinflammation (fibronectin and vitronectin) and neurodegeneration (link protein Hapln2). In fibronectin and Hapln2, this overexpression was more pronounced in aged post-ischemic animals.

**Conclusion:**

Based on these results, we can conclude that the ratio between the protecting and degrading mechanisms in the aged brain is shifted toward degradation and contributes to the aged tissues’ increased sensitivity to ischemic insults. Altogether, our data provide fresh perspectives on the processes underlying ischemic injury in the aging brain and serve as a freely accessible resource for upcoming research.

## 1 Introduction

Ischemic stroke occurs in all age groups. However, its occurrence as well as its associated mortality increase with age, which represents one of the most crucial risk factors for stroke (Benjamin et al., 2018). Even though ischemic stroke is associated with the elderly population, most experimental studies exploring its mechanisms have been performed on young animals (rodents) (Brenneman et al., 2010; Tuo et al., 2017). There are only a few recent studies dealing with ischemic stroke in aged animals. These studies have shown that aging in post-ischemic tissue increases microglial proliferation, decreases cortical neurogenesis, increases inflammatory response and impairs the homeostatic abilities of astrocytes (Moraga et al., 2015; Androvic et al., 2020; Kolenicova et al., 2020). Moreover, age-related processes are accompanied by neuronal changes/loss, followed by glial cell response, increased astrogliosis and microglial activation. These processes can affect the volume of brain extracellular space (ECS) and usually evoke its shrinkage (Syková et al., 2002; Resnick et al., 2003; Cicanic et al., 2018). Smaller ECS thus further increases the concentration of neurotoxic substances released into the ECS. All of these changes can contribute to larger ischemic damage and a worse post-ischemic outcome in aged tissue. Aging is also a critical factor altering brain extracellular matrix (ECM) properties and its expression (Morawski et al., 2014; Reed et al., 2018; Song and Dityatev, 2018; Ewald, 2020; Levi et al., 2020). Alterations in ECM expression, composition, locations or physical properties related to the aging process may thus be an important but yet not fully recognized factor affecting ischemic pathophysiology in aged tissue.

The molecules of the ECM play an important role in many processes mediated by ECM-cell interactions and in supporting the proper function of neurons and glial cells. Cell migration and maturation, differentiation, survival, homeostasis, synaptogenesis, learning and other functions are dependent on ECM components (Morawski et al., 2014; Theocharis et al., 2016; Foscarin et al., 2017; Dzyubenko et al., 2018a). ECM molecules re-modulate cellular plasticity and can either stimulate or suppress immunological and inflammatory responses (Morawski et al., 2014; Deleidi et al., 2015). To better understand ECM functions in physiological and pathological states, it is important to take into consideration which cell type is the source of distinct ECM molecules, as they can be produced by neurons, astrocytes, oligodendrocytes, NG2 glia or mesenchymal cells (for overview see Supplementary Table 1).

The ECM, also generally termed the “matrisome,” represents the ensemble of core ECM and ECM-associated proteins involved in ECM modification and regulation (Naba et al., 2012). The core ECM molecules form three distinct structures: (a) basal membrane (BM), which is composed of collagens and laminins and forms an important border between endothelial and parenchymal tissue as part of the blood-brain barrier (BBB); (b) interstitial matrix, which is a diffuse form of the matrix; and (c) perineuronal nets (PNNs), which are a condensed form of the matrix formed by proteoglycans, link proteins and tenascins (Lau et al., 2013; Fawcett et al., 2019). Adult ECM is largely stable, and alterations in its composition or expression are specific to pathological states or aging (Syková et al., 2002; Foscarin et al., 2017). While certain molecules appear to be neuron-specific under the physiological conditions, in which they are produced, their expression profile may shift toward astrocytes in pathological conditions in response to brain damage or stress, which causes glia activation and neuronal hyperactivity. Under these circumstances, astrocytes and other glial cells may produce neuron-specific PNNs components in sustained quantities (McKeon et al., 1999; Asher et al., 2000; Smith and Strunz, 2005; Schwarzacher et al., 2006). The depletion of oxygen and glucose in the brain alters the molecular processes that lead to the activation of early response genes, major changes in gene expression, and the degradation of ECM components (Edwards and Bix, 2019). ECM-degrading matrix metalloproteinases (MMPs) and several members of the a disintegrin and metalloproteinase with thrombospondin motifs (ADAMTS) family of proteases, are secreted by neurons and glia and drive neural ECM remodeling even in physiological conditions but especially in different pathologies (Malemud, 2006; Gottschall and Howell, 2015). Since ECM complexes are involved in crucial processes affecting brain functions (Dityatev et al., 2010; Song and Dityatev, 2018), the level of their degradation is strictly regulated by tissue inhibitors of metalloproteinases (TIMPs) (Nagase et al., 2006; Brew and Nagase, 2010).

The need for a wider knowledge of ECM modification during aging and ischemia, as well as clarification of the fundamental molecular pathways, has led to transcriptome and proteome investigations in model organisms. Some studies have verified the links between proteins and injury-related cellular processes, as well as evidence for protein interactions involved in neurogenesis and inflammation after stroke (Law et al., 2017; Androvic et al., 2020). The advancement of molecular genetics has made gene interaction studies more accessible. Measuring mRNA expression may aid in explaining the changes in gene expression and their impact on cell characteristics and activities.

This article is based on previously published data (Androvic et al., 2020), which revealed a significant overlap between young adult and aged animals in terms of inflammatory response, cell-cell interaction, and cell cycle progression, as well as their significant upregulation after injury, particularly in aged animals. The current study is a targeted re-analysis of the published dataset, focused on ECM and ECM-modifying proteins. For a better understanding of how the altered gene expression of specific ECM components affects their expression on the protein level, the study was broadened with a proteomic analysis.

## 2 Materials and methods

The datasets that are analyzed in this manuscript, were originally acquired for our previous study and methodical approaches for animal handling, the ischemia model and data acquisition and processing described below are therefore the same (Androvic et al., 2020). A timeline representation of the experimental approaches used in the study is depicted in Supplementary Figure 1.

### 2.1 Animals

Experiments were performed on young adult (3M; 3-month-old) and aged (18M, 18-month-old) C57Black/6. Altogether, 41 female mice were used for this study (24/17 for gene/protein analysis, respectively). The number of mice used in each individual experimental group is indicated in Figure legends. The mice were kept on a 12-h light/dark cycle with access to food and water *ad libitum*. All procedures involving the use of laboratory animals were performed in accordance with the European Communities Council Directive 24 November 1986 (86/609/EEC) and animal care guidelines approved by the Institute of Experimental Medicine, Czech Academy of Sciences (Animal Care Committee on April 7, 2011; approval number 018/2011). All efforts were made to minimize both the suffering and the number of animals used.

### 2.2 Induction of permanent middle cerebral artery occlusion (pMCAo)

Animals were anesthetized with 3% isoflurane (Abbot) and maintained in 2% isoflurane using a vaporizer (Tec-3, Cyprane Ltd.). Between the orbit and the external auditory meatus, a skin incision was made. A 1–2 mm hole was drilled through the frontal bone 1 mm rostrally to the fusion of the zygoma and the squamosal bone and about 3.5 mm ventrally to the dorsal surface of the brain. After the dura was opened and removed, the middle cerebral artery (MCA) was exposed. The MCA was occluded by coagulation with bipolar tweezers (SMT) at a proximal location, followed by transection of the vessel to ensure permanent occlusion. The body temperature was maintained at 37 ± 1°C using a heating pad for the duration of the surgery. This pMCAo model yields small infarct lesions in the parietal cortical region. Intact cortical tissue from 3M and 18M mice was used as a control.

### 2.3 Dissection of brain tissue from the mouse cortex

Mice were deeply anesthetized with pentobarbital (PTB) (100 mg/kg, i.p.), and transcardially perfused with cold (4–8°C) isolation buffer containing (in mM): 136.0 NaCl, 5.4 KCl, 10.0 Hepes, 5.5 glucose, osmolality 290 ± 3 mOsmol/kg. To isolate the cerebral cortex, the brain (+2 mm to −2 mm from the bregma) was sliced into 600 μm coronal sections using a vibrating microtome HM650V (MICROM International GmbH), and the uninjured or post-ischemic parietal cortex was carefully dissected out from the ventral white matter tracts.

### 2.4 RNA isolation, library preparation and sequencing

Tissue-Lyser (Qiagen, USA) was used to homogenize the brain tissue samples. The total quantity of RNA was extracted using TRI Reagent (Sigma-Aldrich, Germany) and the amount and purity was determined using a Thermo Fisher NanoDrop 2000 spectrophotometer. RNA integrity was determined using an Agilent Fragment Analyzer. All samples had RQN > 8. Libraries were prepared from 400 ng total RNA using the Lexogen QuantSeq 3′ Library Prep Kit FWD. 1 μl of ERCC spike-in (c = 0.01x; Thermo Fisher) per library was included. This technique generates stranded libraries that mostly cover the 3′ end of the transcript, resulting in gene-centric expression levels. Libraries were quantified using the Qubit 2 fluorometer (Thermo Fisher) and Fragment Analyzer (Agilent, USA) and sequenced on the NextSeq 500 high-output (Illumina, USA) with 85 bp single-end reads. 11.5–38 million reads were obtained per library with a median of 16 million reads.

### 2.5 RNA-Seq data processing, mapping and counting

TrimmomaticSE v0.36 was used to remove adaptor sequences and low-quality reads (Bolger et al., 2014). SortMeRNA v2.1 with default parameters was used to filter out reads mapping to mtDNA and rRNA (Kopylova et al., 2012). STAR v2.5.2b was used to align the remaining reads to GRCm38 and ERCC reference (Dobin et al., 2013). Mapped reads were counted over Gencode vM8 gene annotation by htseq-count with union mode for the handling of overlapping reads (Anders et al., 2015).

### 2.6 Proteomic analysis

#### 2.6.1 LC/MS sample preparation

Homogenized tissues were lysed in 100 mM TEAB containing 2% sodium deoxycholate, 40 mM chloroacetamide, 10 mM TCEP and boiling at 95°C for 10 min and further sonicated (Bandelin Sonoplus Mini 20, MS 1.5). BCA protein assay kit (Thermo Fisher) was used to determine the protein concentration and 30 μg of protein per sample was used for MS sample preparation. In addition, samples were processed using SP3 beads according to Hughes et al. (2019). For full details see Androvic et al. (2020).

#### 2.6.2 nLC-MS2 analysis

LC/MS analysis was performed by using Nano Reversed phase columns (EASY-Spray column, 50 cm × 75 μm ID, PepMap C18, 2 μm particles, 100 Å pore size). Samples were loaded for 4 min at 18 μl/min onto the trap column (C18 PepMap100, 5 μm particle size, 300 μm × 5 mm, Thermo Fisher). Peptides were eluted with buffer (acetonitrile and 0.1% formic acid) in a gradient from 4 to 35% for 120 min. Eluting peptide cations were converted to gas-phase ions by, and analyzed on, a Thermo Orbitrap Fusion (Q-OT- qIT, Thermo Fisher). Survey scans of peptide precursors from 350 to 1,400 m/z were performed at 120K resolution (at 200 m/z) with a 5 × 10^5^ ion count target. Tandem MS was performed by isolation at 1.5 Th with the quadrupole, HCD fragmentation with normalized collision energy of 30, and rapid scan MS analysis in the ion trap. The MS2 ion count target was set to 10^4^ and the max injection time was 35 ms. Precursors with charge state 2–6 were sampled for MS2. The dynamic exclusion duration was set to 45 s with a 10 ppm tolerance around the selected precursor and its isotopes. Monoisotopic precursor selection was turned on. The instrument was run in top speed mode with 2 s cycles (Hebert et al., 2014).

#### 2.6.3 MS data analysis

Data analysis and quantification was performed with MaxQuant software (version 1.6.3.4) (Cox and Mann, 2008). The false discovery rate (FDR) was set to 1% for both proteins and peptides and the minimum peptide length was seven amino acids. The Andromeda search engine was used for the MS/MS spectra search against the *Mus musculus* database (downloaded from Uniprot on July 2019, containing 22,267 entries). Enzyme specificity was set as C-terminal to Arg and Lys, also allowing cleavage at proline bonds and a maximum of two missed cleavages. Dithiomethylation of cysteine was selected as fixed modification and N-terminal protein acetylation and methionine oxidation as variable modifications. The “match between runs” feature of MaxQuant was used to transfer identifications to other LC-MS/MS runs based on their masses and retention time (maximum deviation 0.7 min) and this was also used in quantification experiments. Quantifications were performed with the label-free algorithm in MaxQuant (Cox and Mann, 2008). Data analysis was performed using Perseus 1.6.1.3 software (Tyanova et al., 2016) and R project v3.6.0. Identifications mapping to more than one protein ID were discarded. The remaining identifications were mapped to gene names based on their ID using the Uniprot database and only uniquely mapping genes were kept for further analysis. The matrix was then further filtered to remove genes with less than 7 positive values and the remaining missing data were imputed according to Wei et al. (2018).

### 2.7 Quantification and statistical analysis

#### 2.7.1 Genes

The results of the experiments were presented as log2Norm.counts (normalized read counts in log2 scale). Differential gene expression from RNA-Seq data was analyzed using DESeq2 v1.16.1150 and the R project. We compared aged controls to young controls, a young stroke group to young controls, an aged stroke group to aged controls, and an aged stroke group to a young stroke group in pairwise comparisons (padj 0.05, log2FC > 1 for upregulation and −0.65 for downregulation). Two-factor comparisons using injury (control/pMCAo) and age (3M/18M) as predictor variables, along with their interactions, were also developed. DESeq2 results are available in the Gene Expression Omnibus repository, accession number GSE137482 or as the Supplementary material within the published article Androvic et al. (2020). The initial analysis of the dataset revealed a high prevalence of genes that were solely induced or repressed in older animals, or with a bigger fold-change. The following process is described in full detail in the article Androvic et al. (2020).

Statistical significance was tested using the Wald test and the *p*-values were adjusted for multiple testing using Benjamini–Hochberg correction. RT-qPCR and MS data were analyzed using linear modeling in R project and GraphPad Prism. Exact sample sizes, significance levels and details of statistical tests and procedures are described in the figure legends.

#### 2.7.2 Proteins

The results of the experiments, available in Supplementary Data Sheet 1, are presented as LFQ intensities (label-free quantification intensities). Statistical analyses of the protein expression were performed by the R-project statistical package (R Core Team, R Foundation for Statistical Computing, Vienna, Austria, http://www.R-project.org; RRID:SCR_001905) and GraphPad Prism. The analysis between the control (uninjured, Ctrl) and the affected (injured, pMCAo) groups was performed using a two-way ANOVA (factors: Age, pMCAo, days after pMCAo). Interaction of the two factors was considered and the two-way ANOVA was followed by Tukey HSD multiple comparisons *post-hoc* test. The values of *p* ≤ 0.05 were considered significant.

## 3 Results

The current study represents a targeted re-analysis of the previously published dataset of age-dependent alterations in the gene expression in response to ischemic brain damage (Androvic et al., 2020). This comprehensive RNA-seq analysis of aging, ischemic stroke, and their interaction was based on 3′mRNA sequencing performed on the parietal cortex isolated from young adult (3M) and aged (18M) female mice, as well as age-matched animals, at 3 days (D3) after pMCAo that corresponds with already developed astrogliosis (in total 4 groups, 6 animals per group). The main results showed downregulation of the axonal and synaptic maintenance genetic program, and increased activation of type I interferon (IFN-I) signaling following stroke in aged mice (Androvic et al., 2020).

Based on our previous studies, revealing that even small shifts in the ratio of particular ECM molecules can affect the protective functions of perineuronal ECM assemblies in the aging processes (Cicanic et al., 2018; Sucha et al., 2020), we hypothesized that altered ECM composition in the aged brain may render the brain more sensitive to ischemic injury and decrease its regeneration ability. To test this hypothesis, we used the dataset generated by Androvic et al. (2020) to explore the genes associated with the proteins of the ECM, and its modifying molecules. Altogether 56 genes of interest were chosen for this study (Supplementary Table 2).

Since mRNA does not always reflect the expression of proteins, the study was expanded by a proteome analysis. A summarized comparison of the gene and protein differential expression is provided as heat maps (Supplementary Figure 2) and described in more detail below.

### 3.1 Transcriptional analysis

#### 3.1.1 Aging

**FIGURE 1:**
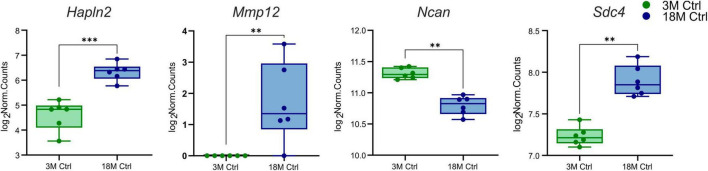
Age-evoked alterations in the gene expression of ECM molecules. Aging revealed significantly changed expression in only four genes from a collection of 56 genes. During aging, the expression of *Hapln2*, *Sdc4*, and *Mmp12* increased, but the expression of *Ncan* decreased. Significance codes: extremely significant (***) for *p* < 0.001; very significant (**) for *p* < 0.01. *N* = 6 animals/group. Statistical methods: Wald test, Benjamini–Hochberg correlation. Ctrl, control, non-ischemic animals; 3M, 3-month-old mice; 18M, 18-month-old mice.

Initially, we concentrated on the genes encoding the ECM molecules that differ in young and aged tissue and which may affect the susceptibility of the brain to ischemic injury. DESeq2 was used to assess the differentially expressed genes of interest between 3 and 18M control mice (Love et al., 2014). Only four ECM genes were found to differ significantly (log_2_FC > 1, *p*_*adj*_ < 0.05) between 3 and 18M tissues—three upregulated genes *Hapln2* (*p*_*adj*_ = 6.29E^–09^), *Sdc4* (*p*_*adj*_ = 0.001), *Mmp12* (*p*_*adj*_ = 0.005) and one downregulated gene *Ncan* (*p*_*adj*_ = 0.005) (Figure 1).

#### 3.1.2 Stroke

**FIGURE 2:**
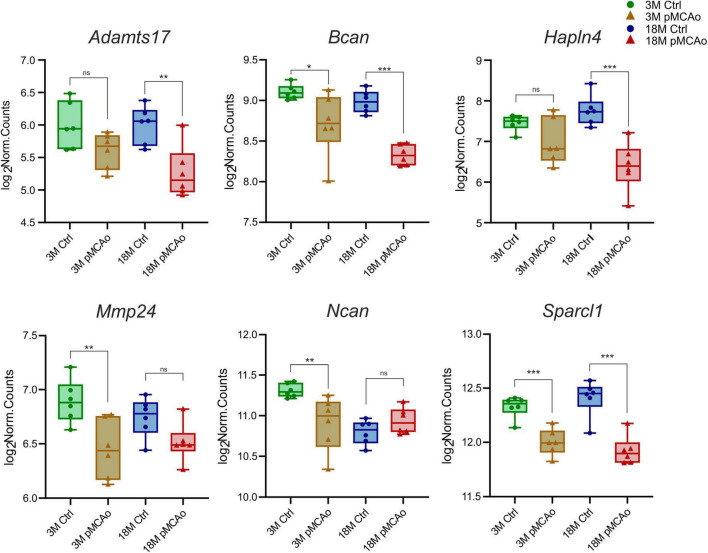
Downregulated ECM genes following stroke in comparison with their age-matched controls. The total number of downregulated genes in young adult (3M) and aged (18M) animals following pMCAo was six. Only two genes (Bcan and Sparcl1) showed significant changes in expression across both age groups. Significance codes: extremely significant (***) for *p* < 0.001; very significant (**) for *p* < 0.01; significant (*) for *p* < 0.05; non-significant (ns) for *p* > 0.05. *N* = 6 animals/group. Statistical methods: Wald test, Benjamini–Hochberg correlation. Ctrl, control, non-ischemic animals; pMCAo, permanent middle cerebral artery occlusion; 3M, 3-month-old mice; 18M, 18-month-old mice.

Furthermore, we focused on comparing transcriptional alterations in 3 and 18M mice D3 after pMCAo with age-matched controls. A significant number of differentially expressed genes of interest were detected in both 3M (39 genes) and 18M (40 genes) mice, with a prevalence of genes that were upregulated following injury (Supplementary Table 3). We revealed only six differentially downregulated genes—in 3M animals: *Bcan* (*p*_*adj*_ = 0.02), *Mmp24* (*p*_*adj*_ = 0.007), *Ncan* (*p*_*adj*_ = 0.008), *Sparcl1* (*p*_*adj*_ = 0.0008); in 18M animals: *Adamts17* (*p*_*adj*_ = 0.0026), *Bcan* (*p*_*adj*_ = 3.3E^–06^), *Hapln4* (*p*_*adj*_ = 8.25E^–07^), *Sparcl1* (*p*_*adj*_ = 6.78E^–08^), see Figure 2.

**FIGURE 3:**
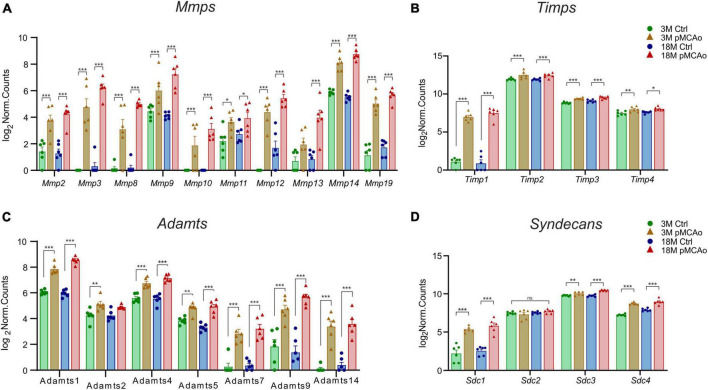
Upregulated ECM genes in D3 after pMCAo in young adult (3M) and aged (18M) mice compared to their corresponding controls. pMCAo influenced the expression of genes belonging to the *Mmp/Timp* complex (10 and 4 genes, respectively) **(A, B)**, to the *ADAMTS* family (7 genes) **(C)** and to the family of syndecans (*Sdc*; 3 genes) **(D)**. Significance codes: extremely significant (***) for *p* < 0.001; very significant (**) for *p* < 0.01; significant (*) for *p* < 0.05; non-significant (ns) for *p* > 0.05. *N* = 6 animals/group. Statistical methods: Wald test, Benjamini–Hochberg correlation. Ctrl, control, non-ischemic animals; pMCAo, permanent middle cerebral artery occlusion; 3M, 3-month-old mice; 18M, 18-month-old mice; Mmps, matrix metalloproteinases; Timps, tissue inhibitors of Mmps.

**FIGURE 4:**
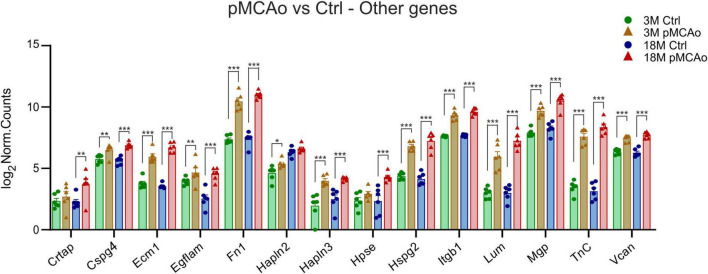
Other ECM upregulated genes in D3 after pMCAo in young adult (3M) and aged (18M) mice compared to their corresponding controls (Ctrl). Significant upregulation in differential expression was also detected in several gene coding proteins assembled in perineuronal nets (*Hapln2*, *3*, *TnC*, *Vcan*) and other genes as associated with the ECM. Significance codes: extremely significant (***) for *p* < 0.001; very significant (**) for *p* < 0.01; significant (*) for *p* < 0.05. *N* = 6 animals/group. Statistical methods: Wald test, Benjamini–Hochberg correlation. Ctrl, control, non-ischemic animals; pMCAo, permanent middle cerebral artery occlusion; 3M, 3-month-old mice; 18M, 18-month-old mice.

We were able to identify a strong upregulation of genes associated with the degradation of the ECM molecules, such as MMPs and their regulating molecules—the TIMPs (Figures 3A, B) and ADAMTS family of ECM degrading enzymes (Figure 3C). Moreover, a significant upregulation of syndecans (Figure 3D) and other genes associated with other ECM molecules, such as proteoglycans, and link proteins, was observed (Figure 4). The *Mmp*/*Timp* complex analysis revealed significant changes in the gene expression of ten Mmp family members (Figure 3A) and all Timp family members (*Timp1-4*) (Figure 3B). In the *Mmp* cluster, 9 genes were elevated in 3M mice and 10 genes in 18M animals. Except for *Mmp13* (upregulation only in 18M, Figure 3A) and *Mmp24* (see downregulated genes, Figure 2), all genes in the cluster were similarly upregulated among age-defined groups (Figure 3A). Of note, the expression of several of the *Mmp(s)* (*Mmp3*, *Mmp10*, and *Mmp12*) in 3 and/or 18M controls was close to the detection limits, whereas pMCAo significantly increased their expression. In both 3 and 18M mice, the whole *Timp* family showed enhanced differential expression following stroke, where alterations in *Timp1* were the most pronounced (*p*_*adj*_ = 5.84E^–25^ 3M; *p*_*adj*_ = 4.99E^–30^ 18M) (Figure 3B). For *p*-values of other members of *Mmp* and *Timp* families see Supplementary Table 3.

Extremely significant upregulation was also found in genes encoding the ADAMTS family of enzymes degrading chondroitin sulphate proteoglycans (CSPGs). This upregulation was mostly detected in post-ischemic animals of both ages (*Adamts1*, *4*, *5*, *7*, *9* and *14*) apart from *Adamts2* which was upregulated only in 3M mice after pMCAo (Figure 3C). Only in one gene of the family (*Adamts 17*) did we find a significant downregulation (Figure 2). For *p*-values see Supplementary Table 3.

According to the analysis of differentially expressed genes, three members of the syndecans family had significantly enhanced gene expression in D3 after pMCAo, both in 3 and 18M tissues: *Sdc1* (*p*_*adj*_ = 4.95E^–11^ 3M, *p*_*adj*_ = 4.35E^–15^ 18M), *Sdc3* (*p*_*adj*_ = 9.71E^–03^ 3M, *p*_*adj*_ = 6.81E^–13^ 18M), and *Sdc4* (*p*_*adj*_ = 1.26E^–23^ 3M, *p*_*adj*_ = 1.68E^–13^ 18M). However, *Sdc2*, which is found in synapses (Hsueh and Sheng, 1999), showed no significant alterations (Figure 3D).

In addition to the aforementioned specific gene families, significant upregulation of the expression of *Cspg4*, *Ecm1*, *Egflam*, *Fn1*, *Hapln3*, *Hspg2* (perlecan), *Itgb1*, *Lum*, *Mgp*, *Tnc* and *Vcan* was detected after pMCAo in both 3 and 18M groups of animals (Figure 4). The expression of some of the examined genes was altered only in 3 or 18M animals. For example, in 3M animals, *Hapln2* (*p*_*adj*_ = 0.01) was significantly upregulated Alternatively, genes *Hpse* (*p*_*adj*_ = 6.61E^–05^) and *Crtap* (*p*_*adj*_ = 0.003) were significantly altered only in 18M animals (Figure 4).

#### 3.1.3 Stroke 3 vs. 18M

**FIGURE 5:**
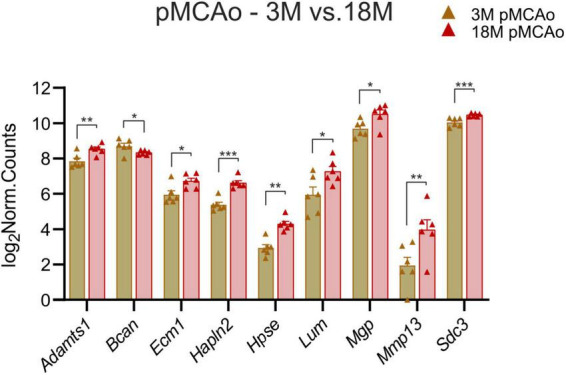
pMCAo. Comparison of ECM gene expression alterations in young adult (3M) and aged (18M) animals after pMCAo. Significant differences between young (3M) and aged (18M) post-ischemic mice were revealed in 9 genes. One of them was downregulated (*Bcan*), the rest were upregulated. Significance codes: extremely significant (***) for *p* < 0.001; very significant (**) for *p* < 0.01; significant (*) for *p* < 0.05. *N* = 6 animals/group. Statistical methods: Wald test, Benjamini–Hochberg correlation. pMCAo, permanent middle cerebral artery occlusion; 3M, 3-month-old mice; 18M, 18-month-old mice.

To identify the genes which could contribute to the increased susceptibility of the aged tissue to ischemic injury, we investigated which ECM genes were expressed differently in 3 and 18M mice following stroke. We found nine genes with significant differences in expression; one of them was downregulated at 18M—*Bcan* (*p*_*adj*_ = 0.018), and eight of them were upregulated at 18M—*Adamts1* (*p*_*adj*_ = 0.0031), *Ecm1* (*p*_*adj*_ = 0.047), *Hapln2* (*p*_*adj*_ = 6.59E^–06^), *Hpse* (*p*_*adj*_ = 0.0026), *Lum* (*p*_*adj*_ = 0.03), *Mgp* (*p*_*adj*_ = 0.01), *Mmp13* (*p*_*adj*_ = 0.008) and *Sdc3* (*p*_*adj*_ = 0.0009) (Figure 5).

### 3.2 Proteomic analysis of the brain ECM

**FIGURE 6:**
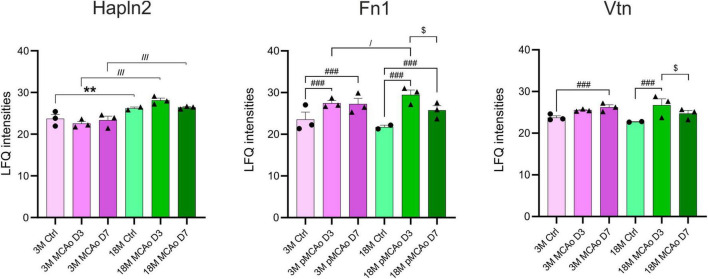
Age- or pMCAo-induced changes in ECM protein expression. Aging on the proteomic level evoked a significant change only in the expression of Hapln2. Ischemia induced significant changes in fibronectin (Fn1) and vitronectin (Vtn) in D3 and D7 following pMCAo in both young as well as aged animals in comparison to their age-matched controls. Moreover, in post-ischemic aged animals, we observed a significant drop of expression of Fn1 and Vtn in D7 compared to D3. Analysis of the differences between young and aged post-ischemic animals revealed significant differences in Hapln2 (in D3 and D7) and Fn1 (only in D3). Significance between 3M Ctrl and 18M Ctrl is marked by asterisk (*), between Ctrl and pMCAo by hashtag (#), between 3M pMCAo and 18M pMCAo by slash (/), and between D3 pMCAo and D7 pMCAo by dollar sign ($). Significance codes: extremely significant (^###^, ^///^) for *p* < 0.001; very significant (**) for *p* < 0.01; significant (^/^, ^$^) for *p* < 0.05. *N* = 3 animals/group; only for 18M Ctrl *N* = 2 animals. Statistical methods: two-way ANOVA, Tukey post-test. Ctrl, control, non-ischemic animals; pMCAo, permanent middle cerebral artery occlusion; 3M, 3-month-old mice; 18M, 18-month-old mice; D3, 3 days after pMCAo; D7, 7 days after pMCAo; LFQ, label-free quantification.

A proteomic analysis was included in the study as the gene expression and protein expression do not necessarily correlate. Therefore, we compared the expression of proteins associated with our genes of interest in 3 and 18M non-operated controls and mice following pMCAo. Compared to the gene analysis that was performed in D3 following pMCAo, samples for the proteomic analysis were collected on days 3 (D3) and 7 (D7) after pMCAo due to a delay in protein production. In our proteomic analysis, we were able to detect only 18 proteins encoded by our set of genes (Supplementary Figure 3). From these 18 proteins we found age- or ischemia-evoked significant changes only in Hapln2, Fn1 and Vtn (Figure 6).

#### 3.2.1 Aging

From our set of proteins, we detected age-related changes only in one protein of the ECM protein group. Link protein Hapln2 was upregulated in 18M animals compared to 3M animals (*p*_*adj*_ = 7.80E^–03^) (Figure 6 and Supplementary Figure 3A), which corresponds with the significantly higher expression of its relevant gene *Hapln2* (Figure 1).

#### 3.2.2 Stroke

Furthermore, we compared proteomic data from 3 and 18M controls with their age-matched animals that underwent pMCAo. Significantly altered expressions were found in Fn1 and Vtn (Figure 6 and Supplementary Figures 3B, C). Compared to non-ischemic age-matched controls, Fn1 expression after pMCAo was significantly higher on D3 as well as on D7 (*p*_*adj*_ < 0.0001) in both 3- and 18M-old animals (Figure 6 and Supplementary Figures 3B, C). In contrast, the expression of Vtn was significantly elevated after pMCAo only on D7 (Vtn, *p*_*adj*_ = 1.90E^–03^) in 3M- old animals and in D3 (*p*_*adj*_ < 0.0001) in 18M-old animals (Figure 6 and Supplementary Figures 3B, C). Moreover, in post-ischemic aged animals, a significant drop of expression was also detected between D3 and D7 in Fn1 (*p*_*adj*_ < 0.0001) and Vtn (*p*_*adj*_ = 0.0205).

#### 3.2.3 Stroke 3 vs. 18M

A comparison of proteomic analysis performed following pMCAo between 3 and 18M animals revealed significant changes in Hapln2 and Fn1. Hapln2 expression was more elevated in aged animals in D3 (*p*_*adj*_ < 0.0001) as well as in D7 (*p*_*adj*_ < 0.0001), which correlates with the differences found in its relevant gene (Figure 5). However, the expression of Fn1 in post-ischemic 18M mice was significantly altered in comparison with similarly affected 3M mice only in D3 (*p*_*adj*_ = 0.0214) (Figure 6 and Supplementary Figure 3D).

## 4 Discussion

Regardless of the type of ischemia, the main mechanisms and processes are relatively well understood. Astrocytes and NG2 glia exhibit characteristic responses to central nervous system (CNS) pathology: rapid cell swelling occurring immediately after ischemia or trauma, and later, reactive gliosis developing within 1–3 days after insult, characterized by proliferation and cellular hypertrophy (Anderova et al., 2004, 2014; Alonso, 2005; Kirdajova et al., 2021). Activated astrocytes are important sources of overexpressed ECM, which participate in the formation of glial scar as well as of proteases that modify the ECM composition. Glial cells are among the major extrinsic factors that facilitate the remodeling of the ECM and PNN, thereby acting as key regulators of the diverse functions of the ECM and PNN in health and disease (Song and Dityatev, 2018; Tewari et al., 2022). Accumulation of reactive astrocytes in the brain is associated with normal aging and may thus contribute to the ECM remodeling associated with aging (Rodriguez et al., 2014).

The aim of our ECM-targeted analysis was to detect variations in the expression of ECM molecules in healthy and post-ischemic brain tissue in young adult (3M) and aged (18M) mice in order to identify the important players affecting the mechanisms of ischemic damage and repair processes, and to explain the elderly brain’s increased susceptibility to ischemic damage. We focused on 56 ECM genes involved in neural regeneration, neurite outgrowth and processes associated with brain damage. Several of them exhibited consistent expression alterations in their differential expression 3 days after pMCAo. Due to a delay caused by biosynthesis, the proteomic analysis was accomplished at two time-points: D3 for comparison with differential gene expression, and D7 for better understanding of the subsequent alterations in the brain, particularly following pMCAo.

**FIGURE 7:**
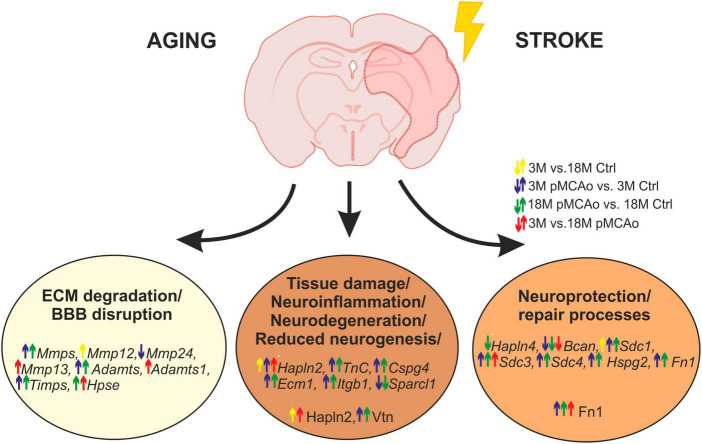
Age or pMCAo induced changes in ECM expression—graphical summary. The figure summarizes the effect of aging and/or ischemia on mRNA or protein expression of selected ECM molecules involved in ECM degradation, neuroinflammation/neurodegeneration and neuroprotection/repair processes. Up- or downregulation of their expression is depicted by arrows (yellow—effect of aging in non-ischemic mice, blue—effect of pMCAo in young adult mice, green–effect of pMCAo in aged mice, red—effect of aging in post-ischemic mice). Expression of genes from *Mmp*, *Timps*, and *Adamts* families were generally upregulated in both young and aged post-ischemic mice except for *Mmp12*, *13*, *24* and *Adamts1*. Genes (in italics) and proteins are divided into specific groups according to their main function. However, due to the multifunctionality of some molecules, it is difficult to assign them as purely beneficial or detrimental, and their function is described in more detail in the Discussion. Ctrl, control, non-ischemic animals; pMCAo, permanent middle cerebral artery occlusion; 3M, 3-month-old mice; 18M, 18-month-old mice; ECM, extracellular matrix; BBB, blood-brain barrier. This figure has been created by help of BioRender Software.

In this study, we observed a huge activation of genes related to ECM degradation, such as *MMPs* following pMCAo, as well as a significant overexpression of *TIMPs*, presumably as a reaction to the *MMP* activation. Moreover, a significant upregulation was also detected in the genes associated with other ECM molecules, such as proteoglycans, syndecans and specific link proteins. Notably, this upregulation in distinct genes was enhanced in old animals, in comparison with the young ones. On the other hand, ischemia evoked a significant downregulation of genes associated with the protective functions of ECM molecules (e.g., *brevican*, *Hapln4*); this downregulation was also more prominent in the aged mice. Our main findings concerning the effect of age and/or ischemia on ECM expression are graphically summarized in Figure 7. Detailed changes in distinct ECM molecules are discussed below.

### 4.1 ECM molecules in perineuronal nets

PNNs are complexes of distinct ECM molecules, specifically lecticans of the CSPG family, tenascins, hyaluronan and hyaluronan-binding link proteins (Hapln1-4) (Bekku et al., 2003; Carulli et al., 2006). It has been shown that ECM, namely PNNs, have a protective function in neurodegeneration and aging (Suttkus et al., 2014; Cicanic et al., 2018; Sucha et al., 2020). PNNs, however, impede nervous system regeneration by inhibiting axonal development (Massey et al., 2006) and terminating synaptic plasticity (Carulli et al., 2010). Partial loss of cortical PNNs associated with the formation of glial scar was reported after transient MCAo by Dzyubenko et al. (2018b). Moreover, using 3D super resolution imaging and mathematical reconstruction of ECM meshworks, the study also revealed alterations in the ultrastructural organization of PNN topology induced by mild hypoperfusion that could hypothetically facilitate local rewiring following stroke (Dzyubenko et al., 2018b). Indeed, restoring the tissue plasticity by enzymatic digestion, or blocking or removal, may affect functional recovery after various brain lesions (for review see Fawcett et al., 2019). Thus, subtle changes in the composition and organization of PNNs might be crucial for balancing their dual function in the development of tissue damage or repair.

Brain-specific link proteins (Hapln1, 2 and 4) are mostly expressed by neurons during development (Supplementary Table 1). Hapln1, the most ubiquitous link protein in the brain, interacts with both hyaluronan and CSPGs, making itself, along with tenascin-R (TnR), a key component of PNNs (Carulli et al., 2010). Hapln1 is involved in the development and initiation of PNNs and the termination of the critical period, and often co-localizes with aggrecan (Acan) (Galtrey et al., 2008; Carulli et al., 2010). Giamanco and Matthews (2012) demonstrated in cell cultures that Acan and hyaluronan are sufficient for base PNN assembly in the absence of glia-derived components Hapln1 and tenascin-R (Giamanco and Matthews, 2012). In contrast, Suttkus et al. (2014) have shown that *Tn-R* and *Hapln1* deficiency has a detrimental impact and results in the loss of neuroprotective characteristics of PNNs (Suttkus et al., 2014). However, on the mRNA level, our findings did not indicate significant differences in *Hapln1* or *Acan* expression in aging or stroke.

Unlike Hapln1, the expression of link proteins Hapln2 (Bral1) and Hapln4 (Bral2) is restricted to distinct brain locations (Oohashi et al., 2002; Bekku et al., 2003, 2010). Hapln2 is associated with the nodal ECM in white matter and can be detected in myelinated fiber tracts extending from the olfactory bulb to the spinal cord (Oohashi et al., 2002). *Hapln2* mRNA expression was upregulated in both young and aged mice following pMCAo. Moreover, the level of *Hapln2* expression significantly increased with aging, not only in controls, but also after pMCAo. *Hapln2* is the only gene where we found significant differences between young and aged post-ischemic tissue and this difference was also detected on the proteomic level. Similar to the *Hapln2* expression, we also found an upregulation of mRNA of versican (Vcan), which often co-localizes with Hapln2 in the nodes of Ranvier of myelinated axons (Bekku et al., 2010). Studies in *Hapln2* knock-outs confirmed its importance for Na^+^ channel clustering in the nodes of Ranvier, which is essential for saltatory conduction and correlates with myelination (Bekku et al., 2010; Susuki et al., 2013). Moreover, studies revealed that Hapln2 overexpression contributes to neurodegeneration in Parkinson’s disease, Alzheimer’s disease as well as schizophrenia, most likely via dysfunction of the ubiquitin-proteasome pathway (UPP) (Lam et al., 2000; Bousman et al., 2010; Shen et al., 2013), which prevents accumulation of potentially toxic proteins within neurons and balances protein synthesis with degradation (for review see Wang et al., 2019). UPP dysfunction occurs with normal aging as well as after cerebral ischemia (Graham and Liu, 2017). Higher upregulation of Hapln2 after pMCAo in aged animals, compared to young ones, may thus be an important factor affecting the extent of the tissue damage and degeneration.

In our study, we also found ischemia-evoked upregulation in *Hapln3*, encoding link protein 3 (LP3). Hapln3/LP3 is widely expressed, mainly outside the brain (Spicer et al., 2003). It was reported to be involved in breast, kidney and prostate cancer (Strand et al., 2014; Wang et al., 2021; Ding et al., 2023) but its role in brain ischemia has not yet been investigated.

The last identified link protein, Hapln4, is expressed only in the distinct nuclei of the brainstem, cerebellum, and thalamus in the mature brain, and in PNNs, it may co-localize with brevican (Bcan) or Acan (Bekku et al., 2003). In this study we found a decreased expression of *Hapln4* gene after pMCAo only in aged mice and downregulation of *Bcan* in both young and aged mice after pMCAo. In addition, the *Bcan* expression was significantly lower in aged mice after pMCAo in comparison to operated young animals. In our previous studies in *Hapln4* knock-out mice, we demonstrated that Hapln4-brevican-based PNNs are crucially important for protection against age-related processes in distinct nuclei of the thalamus and brainstem (Cicanic et al., 2018; Sucha et al., 2020). PNNs formed in Hapln4-deficient tissue, based on Hapln1-Acan or Hapln1-Bcan, were less effective and did not obviate a typical age-related decrease in the extracellular volume or hinder the extracellular diffusion (Cicanic et al., 2018; Sucha et al., 2020). The profound downregulation of *Hapln4* and *Bcan* in the aged mice after pMCAo may thus be another factor affecting ischemic damage in aged tissue.

Neurocan is another CSPG lectin family member, mostly expressed in developing brain tissue by neurons, and its expression decreases significantly within the first month after birth (Rauch et al., 1992; Meyer-Puttlitz et al., 1996). This is in accordance with our finding showing a significant downregulation of *Ncan* in the aged animals. After pMCAo, *Ncan* was downregulated only in the young animals. While previous, mostly *in vitro*, studies have suggested that neurocan plays an important role in axon guidance and neurite growth, Zhou et al. (2001) proposed that it is dispensable for brain development and for the development of hippocampal PNNs (Zhou et al., 2001). However, in the most recent study, Schmidt et al. (2020) demonstrated in the medial nucleus of the trapezoid body that neurocan may influence the mRNA and protein quantity of various PNN molecules and thus contributes to the proper PNN formation and synapse physiology in this nucleus (Schmidt et al., 2020). Studies employing RT-PCR in adult rats after transient MCAo revealed an increased expression of neurocan early after stroke, peaking on D3 or D4 post-stroke and then returning to the basal level (Carmichael et al., 2005; Deguchi et al., 2005). This contrasts with our findings, showing downregulation (young mice) or no change (aged mice) in the *Ncan* gene activity, and no changes in the Ncan protein level after pMCAo. This discrepancy may have arisen from the different models of stroke used in this study—transient versus permanent (our study) MCAo.

The core of PNNs is represented by tenascin-C (TnC) in the immature brain or TnR that prevails in the adult brain. In physiological conditions, TnC is mostly expressed in the developing brain tissue by the radial glia stem cells or is restricted to the stem cell niches in the adult brain, and plays an important role in stem cell proliferation and differentiation (Kazanis et al., 2007; Faissner et al., 2017). Recent findings have suggested that TnC in pathological states may have additional functions, as it is an important inducer of neuroinflammatory cascades, and plays a significant role in the pathogenesis of stroke and brain injury, as well as in the subsequent repair processes (Okada and Suzuki, 2020). Certainly, in our analysis, we detected a strong upregulation of *TnC* expression following pMCAo in both age groups; no changes in *TnR* were detected. Due to the upregulation of TnC following aneurysmal subarachnoid hemorrhage and the possibility to easily measure its concentration in cerebrospinal fluid or plasma by ELISA, TnC was proposed as a biomarker candidate of stroke (Suzuki et al., 2018).

### 4.2 Molecules involved in the enzymatic degradation of ECM

All ECM-based structures, i.e., the BM, interstitial matrix and PNNs, may be modified in various physiological or pathological states by the activity of ECM-degrading enzymes from the MMP or ADAMTS families (Nagase et al., 2006). Almost all cellular types in the CNS (neurons, astrocytes, oligodendrocytes, microglia, endothelial cells) can express MMPs and their regulation protein TIMPs after injury; however, the cellular source may vary according to the specific type of insult (Cunningham et al., 2005). Disintegration of the BM during ischemia has been documented in many investigations (Yepes et al., 2000; Hamann et al., 2004). The BM becomes diffused, thickened and electron-light following ischemia at ultrastructural levels (Kwon et al., 2009; Nahirney et al., 2016). It is anticipated that BM disintegration following stroke is mostly caused by an increased degradation rather than a decreased production of ECM molecules, because the levels and activity of many proteases, including MMPs, are drastically elevated following ischemia (Rosenberg et al., 1996; Gasche et al., 1999; Heo et al., 1999; Rosenberg, 2002).

These studies are in strong agreement with our findings, where we detected a profound upregulation of genes encoding the majority of MMPs, namely *MMP2*, *3*, *8*, *9*, *10*, *11*, *12*, *13*, *14*, and *19*, apart from *MMP24*, which was downregulated in young post-ischemic tissue and not changed in aged animals following pMCAo. Presumably, in reaction to the increased expression of *MMPs*, we also found an upregulation in all four genes encoding their inhibitory proteins, TIMPs.

The activation of MMPs is crucial during the early phases of stroke pathophysiology. MMP2 and MMP9 are the two primary contributors (Klein and Bischoff, 2011). The MMP2, constitutively expressed and secreted as a zymogen, is activated by a complex of TIMP2, pro-MMP2, and MMP14 (membrane-type MMP). On the other hand, the MMP9 production is stimulated by inflammation, and its zymogen cleavage is mostly provided by MMP3 which is triggered during neuroinflammatory responses (Klein and Bischoff, 2011). MMP9 is naturally inhibited by TIMP1 (Vafadari et al., 2016). TIMP2, serving as a damage sensor and aiding in the containment of the injury (Rosenberg et al., 1998; Klein and Bischoff, 2011), can inhibit MMP2 if it is not coupled with MMP14. MMP9 blockage may be detrimental for neurogenic migration and repair processes in the subacute phase (Lee et al., 2006).

It has been shown that the reduction of PNNs in the infarction core, and in other areas containing reactive astrocytes after MCAo, results from the degradation of CSPGs, presumably mediated by ADAMTS activities (Hobohm et al., 2005). Although most ADAMTS are produced by astrocytes, they can also be produced by neurons or microglia (Lemarchant et al., 2013). Due to their inflammatory and anti-angiogenic properties, ADAMTS molecules contribute to the damage/repair balance after ischemic stroke or other brain injury (Gottschall and Howell, 2015). CSPG degradation and axonal regeneration in the brain have been linked in particular to ADAMTS4 (Lemarchant et al., 2016). Cross et al. (2006) reported in 2006, upregulation of ADAMTS1 and 4 following transient middle cerebral artery occlusions in rats detected by western blotting. Indeed, even though we did not detect significant changes in the ADAMTS protein level, we found a profound upregulation of *Adamts1* and *Adamts4* in both post-ischemic age groups. Upregulation after pMCAo was also observed in genes encoding other enzymes of this family: *Adamts 4*, *5*, *7*, *9*, and *14* (in 3 and 18M mice) and in *Adamts2* (only in 3M). The upregulation of *Adamts1* was significantly higher in 18M than in 3M mice, which may imply an increased CSPG degradation in aged post-ischemic animals. In contrast, we found a downregulation of *Adamtsl17* in aged post-ischemic mice compared to age-matched controls.

Upregulation in both age groups after pMCAo was also observed in gene encoding heparanase (*Hpse*), which degrades heparan sulphate proteoglycan (HSPGs). This is in line with other studies showing that heparanase is upregulated in ischemic stroke, expressed in reactive astrocytes and involved in neuroinflammation (Takahashi et al., 2007; Li et al., 2012; Changyaleket et al., 2017).

### 4.3 Heparan sulfate proteoglycans

The HSPGs family includes surface proteins that play important roles in the control of connectivity, axon guidance, and synapse function. They are expressed mostly by astrocytes, although other cellular types may participate (Supplementary Table 1). Previous genomic studies have linked these proteins to neurodevelopmental and neuropsychiatric disorders (Parikshak et al., 2013; De Rubeis et al., 2014). Members of the HSPG family also include glypicans, key factors for excitatory synapse formation in neurons (Allen et al., 2012). Among various HSPG molecules, we detected significant alterations in genes coding syndecans and perlecan (*HSPG2*) in both young and aged animals after pMCAo.

Syndecans are a transmembrane protein family made up of four members, Sdc1-4. They also function as co-receptors for several ECM proteins, including laminin, fibronectin, tenascins, collagen, and others (Elenius et al., 1991; Woods and Couchman, 2001; Dansie and Ethell, 2011). The interaction of syndecans with cytokines, chemokines, growth factors, and ECM components enables their modulation of inflammatory response pathways (Bartlett et al., 2007; Gopal, 2020). Moreover, they are involved in the wound healing and reparation processes following tissue damage so the upregulation of syndecan genes after pMCAo is not surprising. Sdc3 is the most specific syndecan expressed in the brain. *Sdc3* expression in our analysis was certainly the highest, compared to other syndecans. While Sdc3 plays a critical role in neural migration or neurite outgrowth (Raulo et al., 1994; Hienola et al., 2006), Sdc1, 2 and 4 are involved in wound healing, angiogenesis and re-epithelialisation (Elenius et al., 1991; Echtermeyer et al., 2001; Stepp et al., 2002). Kaur et al. (2009) discovered that *Sdc2* expression in the corpus callosum of neonatal rats decreases dramatically with development, but hypoxia increases its expression. Our findings do not support these observations as we found significantly upregulated expression of all syndecans with the exception of *Sdc2*. In both aging and stroke, the expression of *Sdc2* exhibited minimal variations in the mRNA levels. The discrepancy might arise from different locations and the different models used in Kaur’s and our studies: corpus callosum during hypoxia in rats and parietal cortex during pMCAo in mice, respectively.

A huge upregulation following pMCAo in young as well as aged mice was observed in gene *Hspg2*, encoding perlecan. Perlecan is a basement membrane protein and as a component of the vascular ECM, it helps to maintain the endothelial barrier function. Nakamura et al. (2019) have shown that perlecan is upregulated after stroke and is important for BBB maintenance and pericyte accumulation after ischemic stroke. Moreover, recent findings indicate that enzymatic degradation of perlecan, occurring early after stroke, generates the C-terminal domain V (DV) of perlecan, which is beneficial as a neuroprotective molecule and a promoter of post-stroke brain repair (Roberts et al., 2012).

### 4.4 Other genes assembled with the extracellular matrix

*CSPG4* encodes a large surface type I transmembrane core proteoglycan also called neuron-glial antigen 2 (NG2). CSPG4/NG2 was intensively studied in connection with gliomas and other tumors where it induces cell proliferation and migration and its expression correlated with malignancy (Schiffer et al., 2018). Currently, it is considered to be a multifunctional protein involved in various molecular processes through its interaction with more than 40 putative ligands and the concurrent involvement of the ectodomain and cytoplasmic tail (Dimou and Gallo, 2015; Tamburini et al., 2019). CSPG4/NG2 is highly expressed in a certain type of non-neuronal, non-vascular cells called NG2 glia (also polydendrocytes or oligodendrocytes precursor cells) and is widely used as their marker, however, it can also be expressed by some other cell types (Vigano and Dimou, 2016). The large capacity of NG2 glia to proliferate and differentiate into other cellular types is triggered especially under some pathological conditions such as ischemia (Honsa et al., 2012; Kirdajova and Anderova, 2020; Li et al., 2020). NG2 expression in NG2-positive cells is strongly upregulated by inflammation and hypoxia (Ampofo et al., 2017). Therefore, it is not surprising that we detected a large upregulation of *CSPG4* in both age groups after pMCAo. However, we did not observe any significant alterations in its protein expression.

The Extracellular matrix protein 1 (Ecm1) gene has been suggested for involvement in brain disorders associated with vascular development such as migraine, stroke, and cervical arterial dissection (Daghals et al., 2022). This was also confirmed by our results showing extremely significant upregulation of *Ecm1* in both post-ischemic groups in comparison with their age-matched controls. Moreover, its upregulation in 18M mice was higher than in 3M animals, which indicates that this gene can affect the sensitivity of aged tissue toward ischemic damage.

Another upregulated gene in post-ischemic tissue was *Itgb1*, which encodes the integrin beta-1 (Itgb-1) protein. Integrins have been shown to be necessary for leukocyte adhesion and migration, and thus they are of interest in many inflammation-associated disorders, including ischemic stroke. Igtb-1 was shown to affect vascular angiogenesis following ischemic stroke. Therefore, it was suggested as critically involved in functional deficits and survival following a stroke (Lathia et al., 2010). Lathia et al. (2010) also showed that the Itgb-1 is upregulated in cerebral vascular endothelial cells several days after stroke and its inhibition suppresses post-stroke cerebral angiogenesis and worsens functional and infarct outcomes (Lathia et al., 2010).

Other heterogeneous groups of genes, where we observed significant alterations after pMCAo, were genes encoding lumican (Lum), pikachurin or EGF like, fibronectin type III and laminin G domains (Egflam), Cartilage-associated protein (Crtap), and matrix gla protein (Mgp). They are mostly expressed by mesenchymal cells and involved in processes outside of the brain: Lum in the organization of collagen fibrils in the cornea, skin and tendon stiffness (Chakravarti, 2002), Egflam is a dystroglycan-interacting protein in the retina (Sato et al., 2008), and Crtap plays a role in connective tissue and skeletal development (Morello and Rauch, 2010). Mgp, a vitamin K-dependent protein, is traditionally considered to inhibit the calcification of arteries and cartilage, even though some results are controversial (Barrett et al., 2018). On the other hand, Mertsch et al. (2009) reported an overexpression of Mgp in glioblastoma cells and suggested that Mgp promotes glioma migration and is associated with a worse outcome (Mertsch et al., 2009). However, the function of these proteins in brain ischemia is not fully investigated yet. We showed that the genes of these proteins were significantly upregulated either in both post-ischemic age groups (*Lum*, *Egflam*, *Mgp*) or at least in aged post-ischemic mice (*Crtap*). Moreover, in *Lum* and *Mgp*, we observed that this upregulation in aged animals is stronger than in young adults. Therefore, it could be worth focusing new studies also on the role of these genes/proteins in brain ischemia.

The SPARC (secreted protein acidic and rich in cysteine) family of proteins is involved in tissue development and repair. These proteins are widely expressed in the CNS of healthy people and their expression is significantly increased in the brain tissue following disease or injury (Chen et al., 2020). SPARC-like protein 1 (SPARCL1 or SC1), is an anti-adhesive glycoprotein with a high structural similarity to SPARC. It dynamically interacts with ECM molecules affecting the ECM-cell adhesion and is considered to be a matricellular protein (Sullivan et al., 2006). SPARCL1 is highly expressed by astrocytes during CNS development and following acute CNS damage (Bridel et al., 2018). Lively et al. (2011) reported that after focal striatal ischemic infarction in adult rats, SPARCL1 is more abundant in the astrocytes surrounding the infarct and in the glial scar, while in aged rats, the expression of SPARCL1 was lower at the lesion edge (Lively et al., 2011). The same group proposed SPARCL1 as a novel early marker of white matter damage in 3 models of acute injury in the rat striatum: transient focal ischemia, intracerebral hemorrhage, and a needle penetration wound (Lively et al., 2011). In contrast to their findings, we did not detect a significant alteration in the protein expression of SPARCL1 after pMCAo, neither in young nor aged animals. However, at the mRNA expression level, we found a significant downregulation of *Sparcl1* in aged animals after pMCAo in comparison with their age-matched controls. Nevertheless, the results of Lively et al. (2011) and our studies cannot be fully comparable due to different models (transient focal ischemia induced by endothelin 1 in rats vs. pMCAo in mice, respectively) and the different detection methods (immunohistochemistry and image analysis vs. liquid chromatography/mass spectrometry, respectively) used.

### 4.5 Proteomic analysis

Besides Hapln2 that was discussed above, our analysis of protein expression alterations following ischemia revealed significant changes in the expression of two essential molecules, Fn1 and Vtn. Previous studies have demonstrated that these proteins have a considerable direct influence on the promotion of microglial activation (Milner and Campbell, 2003; Jia et al., 2020) and MMP9 production (Milner et al., 2007). Fn1 and Vtn were found at extremely low concentrations in the normal adult CNS (Risau and Lemmon, 1988; Venstrom and Reichardt, 1993; Milner et al., 2007).

Fibronectin is a large glycoprotein that exists especially in a soluble form as a plasma protein and is produced by endothelial cells. In the CNS, fibronectin is produced and released by neuroglial cells and assembled into the ECM (Tsuda et al., 2008; Lin et al., 2012). Its function in the brain involves the modulation of neurotrophic and anti-inflammatory processes, the axon growth and neuron survival (Duan et al., 2000; Neiiendam et al., 2004; Hansen et al., 2008). It has been shown that Fn1 plays an important role in the pathogenesis of brain injury (Tate et al., 2007b; George and Geller, 2018). Fn1 enters the brain parenchyma together with macrophages as a result of brain injury, and the breakdown of the BBB and activates the microglia and macrophages responsible for clearing the debris following injury (Tate et al., 2007b; George and Geller, 2018). It was suggested that Fn1 has a neuroprotective role following brain injury (Tate et al., 2007a). Certainly, in studies on spinal cord injury (King et al., 2006) and in cultured oligodendrocytes (Hu et al., 2009; King et al., 2010), fibronectin had an enhancing effect on axon growth, reduction of the lesion and apoptosis. In our analysis, the differential expression of *Fn1* after pMCAo revealed a significant upregulation in young and aged mice in comparison to their age-matched controls. These results also agree with the Fn1 protein expression. A significantly increased level of Fn1 in young and aged mice after stroke can be either a result of an inflow of the soluble form of Fn1 from plasma or caused by the higher production of Fn1, which can be related with the upregulation of *Fn1*. In addition, the analysis revealed a rapid increase in Fn1 protein expression on D3 after pMCAo in young adult and aged animals. On D7 after pMCAo, we observed a significant decrease in Fn1 expression in aged animals in comparison to D3. These data may imply that in aged tissue, Fn1 plays a more active role in the acute tissue response after focal cerebral ischemia.

In contrast to Fn1, the level of Vtn protein was significantly increased only in the aged animals. Since our gene analysis did not reveal any changes in the expression of *Vtn*, we assume that the increased level of Vtn protein is a result of its relocation to the brain. Vtn is a protein produced by the liver and, similarly to Fn1, it leaks into the blood and transfers to the brain after injury (Jia et al., 2019). Vtn can bind to integrin receptors and activate signaling mediators (Giancotti and Ruoslahti, 1999; Humphries et al., 2006). Studies on baboons provide evidence about the deposition of Vtn in the brain after stroke in males, but not in females, and about its activation of microglia (del Zoppo et al., 2012). These changes contribute, together with the infiltration of macrophages, to detrimental effects on brain tissue by the development of inflammation and production of cytokines and MMPs (del Zoppo et al., 2012; Taylor and Sansing, 2013). A recent study published by Jia et al. (2020) showed that Vtn is transferred from the bloodstream into the injured brain after stroke and reduces neurogenesis by the overexpression of stroke-induced IL-6, but only in the female brain. The sexual dimorphism in the Vtn effect on neurogenesis is caused by a different level in the expression of IL-6 at D1 after stroke, and its reduction to the normal level lasts for 2–3 days (Berti et al., 2002; Kang et al., 2013). These findings are important in the context of our study, which was conducted on female mice.

Milner et al. (2007) published an interesting study about microglial activation influenced by Fn and Vtn and mediated by the integrins α_5_β_1_ and α_V_β_5_. These findings represent a promising discovery for the development of therapeutic approaches aimed at the inhibition of microglial activation, which is associated with brain diseases and injuries. Due to their ability to reverse the structural and functional alterations of the ECM, Fn1 and Vtn may be considered as promising clinical tools for the treatment of neurodegenerative disorders.

It has to be stated that we were only able to detect 18 proteins encoded by our set of 56 genes related to ECM in our proteomic analysis. The original study of Androvic et al. (2020) did not focus primarily on the ECM molecules and for tissue solubilization for proteomic analysis, a standard protocol was therefore used. However, several studies showed that some proteins, in particular the ECM, may resist standard protocols for solubilization. For example, using a consecutive extraction with four distinct detergent mixtures, Kjell et al. (2020) identified 4 groups of ECM proteins with different solubility profiles (Kjell et al., 2020). Our analysis might therefore miss some important ECM proteins, especially from the more soluble fractions, that could be identified using different tissue processing protocols.

## 5 Conclusion

Regardless of the number of studies dealing with the function of ECM in brain pathologies, its role in the process of how aging may affect ischemic pathophysiology, the extent of the tissue damage and the subsequent repair processes, is not yet fully elucidated. Reflecting on the observed changes in the gene/protein expression of the ECM molecules, we conclude that the ratio between protection and degradation in the aged brain is shifted toward degradation, and contributes to the aged tissues’ increased sensitivity to ischemic insults. A particular advantage of this study is the identification of 9 genes and two proteins of ECM with a significantly altered expression between young and aged post-ischemic mice, whose possible involvement in a more severe outcome of ischemia in aged tissue could be worth focusing on in the future.

We are aware of the potential limitations of our study, resulting from an investigation performed purely on female mice and on a single time-point design (D3) for gene analysis, and a two-time-point design (D3, D7) for protein analysis. With the exception of Hapln2 and Fn1, we were not able to detect similar changes in the expression levels of genes and their relevant proteins, possibly due to the limited sample size used for proteomic analysis or due to the standard protocol used for tissue processing. Despite its limitations, we believe that our study can add to a better understanding of age-related modifications of ischemic mechanisms and will inspire further investigations that may pave new directions in pathophysiology of ischemia and post-ischemic regeneration.

## Data availability statement

The gene data presented in this study are deposited in the Gene Expression Omnibus repository, accession number GSE137482 In addition, processed gene data are available as Supplementary material within the article: Androvic et al. (2020). The data concerning the protein expression are included as Supplementary Data Sheet 1 within this article.

## Ethics statement

The animal study was approved by the Animal Care Committee of the Institute of Experimental Medicine, Czech Academy of Sciences. The study was conducted in accordance with the local legislation and institutional requirements.

## Author contribution

MC: Data curation, Formal analysis, Investigation, Visualization, Writing – original draft, Writing – review and editing. PA: Writing – review and editing, Data curation, Formal analysis, Investigation. DK: Data curation, Formal analysis, Investigation, Writing – review and editing. JT: Data curation, Investigation, Writing – review and editing. JK: Data curation, Investigation, Writing – review and editing. LuV: Conceptualization, Supervision, Validation, Writing – review and editing. MA: Conceptualization, Funding acquisition, Supervision, Validation, Writing – review and editing. LyV: Conceptualization, Supervision, Validation, Writing – original draft, Writing – review and editing.
