# Supplementary material for: A view of the genetic and proteomic profile of extracellular matrix molecules in aging and stroke
Source: Front Cell Neurosci. 2023 Nov 30;17:1296455. doi: 10.3389/fncel.2023.1296455 (PMC10723838; doi:10.3389/fncel.2023.1296455)
Supplement: Supplementary file 1 [file Image_1.PDF]

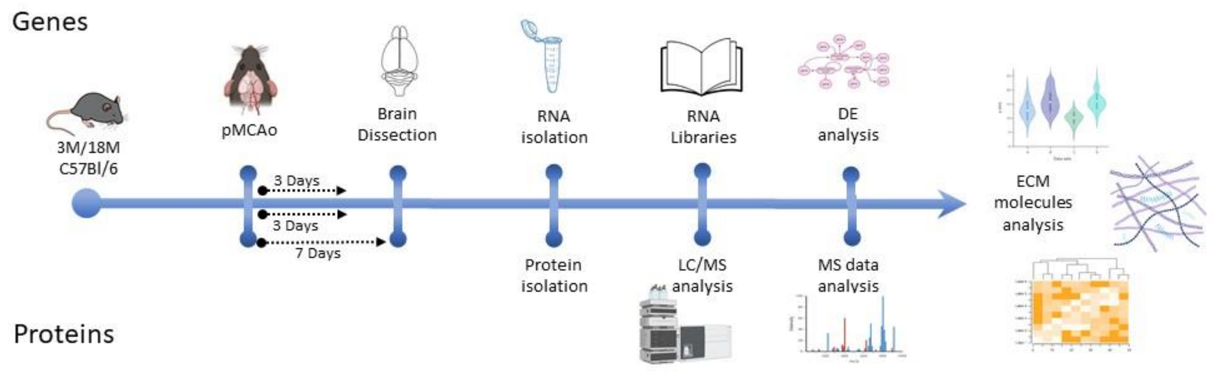

Supplementary Figure 1: **Timeline of the experimental approach.** Created with BioRender.com. **Abbreviations:** 3M (3-month-old mice), 18M (18-month-old mice), pMCAo (permanent middle cerebral artery occlusion), DE (differential expression), LC (liquid chromatography), MS (mass spectrometry), ECM (extracellular matrix).
