# Supplementary material for: A view of the genetic and proteomic profile of extracellular matrix molecules in aging and stroke
Source: Front Cell Neurosci. 2023 Nov 30;17:1296455. doi: 10.3389/fncel.2023.1296455 (PMC10723838; doi:10.3389/fncel.2023.1296455)
Supplement: Supplementary file 2 [file Image_2.PDF]

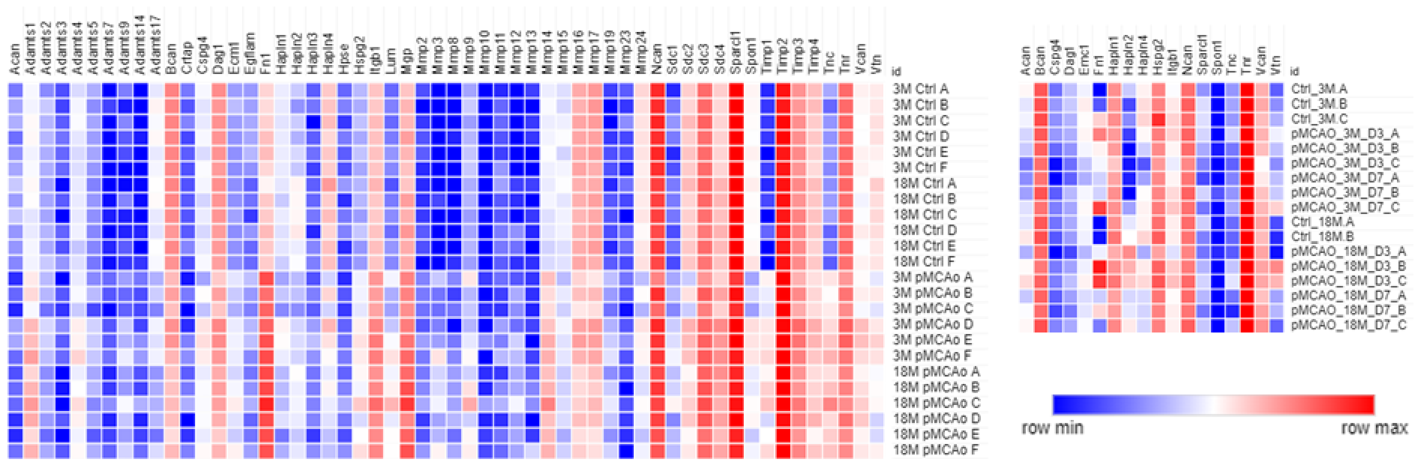

Supplementary Figure 2: Heatmaps of the differential gene (left) and protein expression (right) in young adult (3M) and aged (18M) controls and mice after pMCAo. In total, 56 genes and 18 proteins were observed. Gene expression was analyzed on D3; protein expression was evaluated on D3 and D7. D3, D7 - the third and seventh day after pMCAo, respectively. N = 6 animals/group for genes and N = 3 animals/group for proteins, only in 18M Ctrl N = 2 animals. Statistical methods: Wald test, Benjamini-Hochberg correlation for genes, Two-way ANOVA, Tukey post-test for proteins. **Abbreviations:** Ctrl (control, non-ischemic animals), pMCAo (permanent middle cerebral artery occlusion), 3M (3-month-old mice), 18M (18-month-old mice), D3 (three days after pMCAo), D7 (seven days after pMCAo).
