# Supplementary material for: A view of the genetic and proteomic profile of extracellular matrix molecules in aging and stroke
Source: Front Cell Neurosci. 2023 Nov 30;17:1296455. doi: 10.3389/fncel.2023.1296455 (PMC10723838; doi:10.3389/fncel.2023.1296455)
Supplement: Supplementary file 3 [file Image_3.PDF]

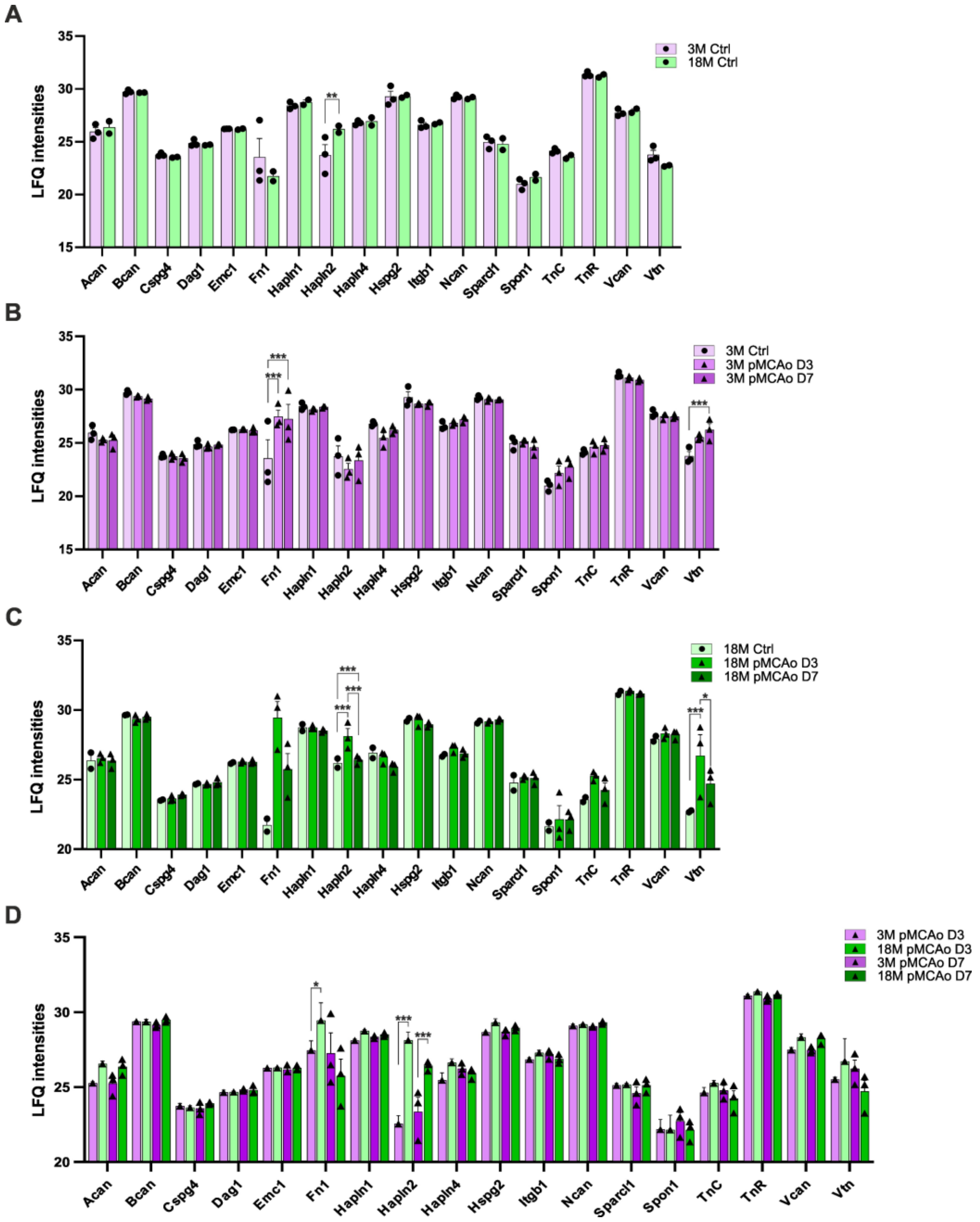

Supplementary Figure 3: Comparison of protein expression in aging (A), after ischemia in young adult (B) or in aged (C) mice and between young and aged post-ischemic animals (D). Aging itself was accompanied by only one significant change in protein expression - Hapln2 (A). Ischemia induced a significantly increased expression of fibronectin (Fn1) and vitronectin (Vtn) in young adult (B), as well as in aged animals (C). The comparison of young and aged post-ichemic mice showed significant alterations in Hapln2 and Fn1. For more detailed analysis of differential expression Hapln2, Fn1 and Vtn changes see also Figure 6 in the main text. Significance codes: extremely significant (***) for $p < 0.001$; very significant (**) for $p < 0.01$; significant (*) for $p < 0.05$; non-significant (ns) for $p > 0.05$. N = 3 animals/group; only for 18M Ctrl N = 2 animals/group. Statistical method: Two-way ANOVA, Tukey post-test. **Abbreviations:** Ctrl (control, non-ischemic animals), pMCAo (permanent middle cerebral artery occlusion), 3M (3-month-old mice), 18M (18-month-old mice), D3 (three days after pMCAo), D7 (seven days after pMCAo), LFQ (label-free quantification).
