# Supplementary material for: A view of the genetic and proteomic profile of extracellular matrix molecules in aging and stroke
Source: Front Cell Neurosci. 2023 Nov 30;17:1296455. doi: 10.3389/fncel.2023.1296455 (PMC10723838; doi:10.3389/fncel.2023.1296455)
Supplement: Supplementary file 4 [file Table_1.PDF]

Supplementary Table 1: Cellular sources of the major extracellular matrix components. Astrocytes, neurons, endothelial cells (ECs), oligodendrocytes (OLs) and their precursors (OPCs) provide molecules to build perineuronal nets.
Abbreviations: CSPGs (chondroitine sulfate proteoglycans), HSPGs (heparan sulfate proteoglycans), LP (link proteins), ECs (endothelial cells), OLs (oligodendrocytes), OPCs (oligodendrocyte precursor cells).

ECM molecules		Cellular source	Citation
**CSPGs**	Aggrecan	Astrocytes Neurons	[1-6]
	Brevican	Astrocytes Neurons OLs	[1, 7]
	Neurocan	Astrocytes Neurons	[1, 3, 8, 9]
	Versican	Astrocytes Neurons OLs NG2	[1, 3, 10]
**HSPGs**	Syndecans	Astrocytes Neurons OLs and OPCs Microglia	[12-15]
	Perlecan	Astrocytes OLs and ECs Microglia	[16-18]
**LP**	Hapln1	Neurons	[1, 5, 23]
	Hapln2	Neurons OLs	[1, 5, 23, 24]
	Hapln4	Neurons	[5]
**Fibronectin**		ECs Pericytes Macrophages	[25]
**Tenascin-C**		Astrocytes Neurons OLs	[25-27]
**Tenascin-R**		Astrocytes Neurons OLs	[1, 5, 6, 23, 28]
**Adamts**		Astrocytes Neurons Microglia	
**MMPs/Timps**		Astrocytes Neurons OLs Microglia	[29]

1. Carulli, D., et al., *Composition of perineuronal nets in the adult rat cerebellum and the cellular origin of their components.* J Comp Neurol, 2006. **494**(4): p. 559-77.
2. Miyata, S., et al., *Construction of perineuronal net-like structure by cortical neurons in culture.* Neuroscience, 2005. **136**(1): p. 95-104.
3. Abaskharoun, M., et al., *Expression of hyaluronan and the hyaluronan-binding proteoglycans neurocan, aggrecan, and versican by neural stem cells and neural cells derived from embryonic stem cells.* Brain Res, 2010. **1327**: p. 6-15.
4. Afshari, F.T., et al., *Schwann cell migration is integrin-dependent and inhibited by astrocyte-produced aggrecan.* Glia, 2010. **58**(7): p. 857-69.
5. Galtrey, C.M., et al., *Distribution and synthesis of extracellular matrix proteoglycans, hyaluronan, link proteins and tenascin-R in the rat spinal cord.* Eur J Neurosci, 2008. **27**(6): p. 1373-90.
6. Giamanco, K.A., M. Morawski, and R.T. Matthews, *Perineuronal net formation and structure in aggrecan knockout mice.* Neuroscience, 2010. **170**(4): p. 1314-27.
7. Hamel, M.G., J. Mayer, and P.E. Gottschall, *Altered production and proteolytic processing of brevican by transforming growth factor beta in cultured astrocytes.* J Neurochem, 2005. **93**(6): p. 1533-41.
8. Margolis, R.K., et al., *Neurocan and phosphacan: two major nervous tissue-specific chondroitin sulfate proteoglycans.* Perspect Dev Neurobiol, 1996. **3**(4): p. 273-90.
9. Asher, R.A., et al., *Neurocan is upregulated in injured brain and in cytokine-treated astrocytes.* J Neurosci, 2000. **20**(7): p. 2427-38.
10. Asher, R.A., et al., *Versican is upregulated in CNS injury and is a product of oligodendrocyte lineage cells.* Journal of Neuroscience, 2002. **22**(6): p. 2225-2236.
12. Kaur, C., et al., *Expression of syndecan-2 in the amoeboid microglial cells and its involvement in inflammation in the hypoxic developing brain.* Glia, 2009. **57**(3): p. 336-49.
13. Iseki, K., et al., *Increased syndecan expression by pleiotrophin and FGF receptor-expressing astrocytes in injured brain tissue.* Glia, 2002. **39**(1): p. 1-9.

14. Properzi, F., et al., *Heparan sulphate proteoglycans in glia and in the normal and injured CNS: expression of sulphotransferases and changes in sulphation.* Eur J Neurosci, 2008. **27**(3): p. 593-604.
15. Sugimoto, K., et al., *Activated microglia in a rat stroke model express NG2 proteoglycan in peri-infarct tissue through the involvement of TGF-beta1.* Glia, 2014. **62**(2): p. 185-98.
16. Garcia de Yebenes, E., et al., *Regulation of the heparan sulfate proteoglycan, perlecan, by injury and interleukin-1alpha.* J Neurochem, 1999. **73**(2): p. 812-20.
17. Saku, T. and H. Furthmayr, *Characterization of the major heparan sulfate proteoglycan secreted by bovine aortic endothelial cells in culture. Homology to the large molecular weight molecule of basement membranes.* J Biol Chem, 1989. **264**(6): p. 3514-23.
18. Winkler, S., et al., *Syndecan-3 and perlecan are differentially expressed by progenitors and mature oligodendrocytes and accumulate in the extracellular matrix.* J Neurosci Res, 2002. **69**(4): p. 477-87.
23. Carulli, D., et al., *Animals lacking link protein have attenuated perineuronal nets and persistent plasticity.* Brain, 2010. **133**(Pt 8): p. 2331-47.
24. Oohashi, T., et al., *Bral1, a brain-specific link protein, colocalizing with the versican V2 isoform at the nodes of Ranvier in developing and adult mouse central nervous systems.* Mol Cell Neurosci, 2002. **19**(1): p. 43-57.
25. Siddiqui, S., A. Horvat-Brocker, and A. Faissner, *The glia-derived extracellular matrix glycoprotein tenascin-C promotes embryonic and postnatal retina axon outgrowth via the alternatively spliced fibronectin type III domain TNfnD.* Neuron Glia Biol, 2008. **4**(4): p. 271-83.
26. Siddiqui, S., A. Horvat-Broecker, and A. Faissner, *Comparative screening of glial cell types reveals extracellular matrix that inhibits retinal axon growth in a chondroitinase ABC-resistant fashion.* Glia, 2009. **57**(13): p. 1420-38.
27. Ferhat, L., et al., *Transient increase of tenascin-C in immature hippocampus: astroglial and neuronal expression.* J Neurocytol, 1996. **25**(1): p. 53-66.
28. Giamanco, K.A. and R.T. Matthews, *Deconstructing the perineuronal net: cellular contributions and molecular composition of the neuronal extracellular matrix.* Neuroscience, 2012. **218**: p. 367-84.
29. Cunningham, L.A., M. Wetzel, and G.A. Rosenberg, *Multiple roles for MMPs and TIMPs in cerebral ischemia.* Glia, 2005. **50**(4): p. 329-339.
