# Supplementary material for: A view of the genetic and proteomic profile of extracellular matrix molecules in aging and stroke
Source: Front Cell Neurosci. 2023 Nov 30;17:1296455. doi: 10.3389/fncel.2023.1296455 (PMC10723838; doi:10.3389/fncel.2023.1296455)
Supplement: Supplementary file 5 [file Table_2.PDF]

Supplementary Table 2: List of ECM genes and proteins of interest. Totally, 56 genes and 18 proteins were analysed.

Genes	Proteins	Name	Genes	Proteins	Name
Acan	**Acan**	**Aggrecan core protein**	***Mmp8***		Matrix metalloproteinases 8
Adamts1		A disintegrin and metalloproteinase with thrombospondin motifs 1	***Mmp9***		Matrix metalloproteinases 9
Adamts2		A disintegrin and metalloproteinase with thrombospondin motifs 2	***Mmp10***		Matrix metalloproteinases 10
Adamts3		A disintegrin and metalloproteinase with thrombospondin motifs 3	***Mmp11***		Matrix metalloproteinases 11
Adamts4		A disintegrin and metalloproteinase with thrombospondin motifs 4	***Mmp12***		Matrix metalloproteinases 12
Adamts5		A disintegrin and metalloproteinase with thrombospondin motifs 5	***Mmp13***		Matrix metalloproteinases 13
Adamts7		A disintegrin and metalloproteinase with thrombospondin motifs 7	***Mmp14***		Matrix metalloproteinases 14
Adamts9		A disintegrin and metalloproteinase with thrombospondin motifs 9	***Mmp15***		Matrix metalloproteinases 15
Adamts14		A disintegrin and metalloproteinase with thrombospondin motifs 14	***Mmp16***		Matrix metalloproteinases 16
Adamts17		A disintegrin and metalloproteinase with thrombospondin motifs 17	***Mmp17***		Matrix metalloproteinases 17
Bcan	**Bcan**	Brevican core protein	***Mmp19***		Matrix metalloproteinases 19
Crtap		Cartilage-associated protein	***Mmp23***		Matrix metalloproteinases 23
Cspg4	**Cspg4**	Chondroitin sulfate proteoglycan 4	***Mmp24***		Matrix metalloproteinases 24
Dag1	**Dag1**	Dystroglycan 1	***Ncan***	**Ncan**	Neurocan core protein
Ecm1	**Ecm1**	Extracellular matrix protein 1	***Sdc1***		Syndecan 1
Egflam		Pikachurin	***Sdc2***		Syndecan 2
Fn1	**Fn1**	Fibronectin	***Sdc3***		Syndecan 3
Hapln1	**Hapln1**	Hyaluronan and proteoglycan link protein 1	***Sdc4***		Syndecan 4
Hapln2	**Hapln2**	Hyaluronan and proteoglycan link protein 2	***Sparcl1***	**Sparcl1**	SPARC-like protein 1
Hapln3		Hyaluronan and proteoglycan link protein 3	***Spon1***	**Spon1**	Spondin-1
Hapln4	**Hapln4**	Hyaluronan and proteoglycan link protein 4	***Timp1***		Tissue inhibitor of metalloproteinases 1
Hpse		Heparanase	***Timp2***		Tissue inhibitor of metalloproteinases 2
Hspg2	**Hspg2**	Basement membrane-specific heparan sulfate proteoglycan core protein	***Timp3***		Tissue inhibitor of metalloproteinases 3
Itgb1	**Itgb1**	Integrin beta-1	***Timp4***		Tissue inhibitor of metalloproteinases 4
Lum		Lumican	***TnC***	**TnC**	Tenascin-C
Mgp		Matrix Glia protein	***TnR***	**TnR**	Tenascin-R
Mmp2		Matrix metalloproteinases 2	***Vcan***	**Vcan**	Versican core protein
Mmp3		Matrix metalloproteinases 3	***Vtn***	**Vtn**	Vitronectin
