# Supplementary material for: A view of the genetic and proteomic profile of extracellular matrix molecules in aging and stroke
Source: Front Cell Neurosci. 2023 Nov 30;17:1296455. doi: 10.3389/fncel.2023.1296455 (PMC10723838; doi:10.3389/fncel.2023.1296455)
Supplement: Supplementary file 6 [file Table_3.PDF]

Supplementary Table 3: List of ECM genes with significantly changed expression evoked by aging, ischemia (pMCAo) in young adult (3M) and aged (18M) mice or between groups following pMCAo. Significance values are supplemented with information about increased (up, upregulation) or decreased (down, downregulation) expression. N = 6 animals/group. Statistical method: Wald test, Benjamini-Hochberg correlation. **Abbreviations:** Ctrl (control, non-ischemic tissue), pMCAo (permanent middle cerebral artery occlusion), 3M (3-month-old mice), 18M (18-month-old mice), *ns* (not significant difference).

Gene	Ctrl 3M vs. 18M		3M pMCAo vs. Ctrl		18M pMCAo vs. Ctrl		pMCAo 3M vs. 18M	
Adamts1	*ns*		7.53E^{-22}	**up**	1.33E^{-42}	**up**	3.09E^{-03}	**up**
Adamts2	*ns*		2.38E^{-03}	**up**	*ns*	**up**	*ns*	
Adamts4	*ns*		2.88E^{-08}	**up**	2.55E^{-14}	**up**	*ns*	
Adamts5	*ns*		1.79E^{-03}	**up**	1.98E^{-07}	**up**	*ns*	
Adamts7	*ns*		1.51E^{-04}	**up**	7.42E^{-05}	**up**	*ns*	
Adamts9	*ns*		2.77E^{-07}	**up**	2.16E^{-13}	**up**	*ns*	
Adamts14	*ns*		8.59E^{-06}	**up**	9.21E^{-06}	**up**	*ns*	
Adamts17	*ns*		*ns*		2.55E^{-03}	**down**	*ns*	
Bcan	*ns*		2.16E^{-02}	**down**	3.30E^{-06}	**down**	1.83E^{-02}	**down**
Crtap	*ns*		*ns*		2.76E^{-03}	**up**	*ns*	
Cspg4	*ns*		1.47E^{-03}	**up**	1.90E^{-08}	**up**	*ns*	
Ecm1	*ns*		7.22E^{-14}	**up**	1.20E^{-25}	**up**	4.73E^{-02}	**up**
Egflam	*ns*		2.92E^{-02}	**up**	2.99E^{-05}	**up**	*ns*	
Fn1	*ns*		7.92E^{-33}	**up**	6.36E^{-40}	**up**	*ns*	
Hapln2	6.29E^{-09}	**up**	1.05E^{-02}	**up**	*ns*		6.56E^{-06}	**up**
Hapln3	*ns*		7.50E^{-06}	**up**	6.28E^{-05}	**up**	*ns*	
Hapln4	*ns*		*ns*		8.25E^{-07}	**down**	*ns*	
Hpse	*ns*		*ns*		6.61E^{-05}	**up**	2.65E^{-03}	**up**
Hspg2	*ns*		2.84E^{-17}	**up**	7.17E^{-27}	**up**	*ns*	
Itgb1	*ns*		3.45E^{-27}	**up**	2.14E^{-34}	**up**	*ns*	
Lum	*ns*		6.67E^{-13}	**up**	9.02E^{-23}	**up**	3.31E^{-02}	**up**
Mgp	*ns*		5.33E^{-12}	**up**	8.42E^{-19}	**up**	1.11E^{-02}	**up**
Mmp2	*ns*		2.57E^{-05}	**up**	5.29E^{-07}	**up**	*ns*	
Mmp3	*ns*		5.05E^{-11}	**up**	6.76E^{-11}	**up**	*ns*	
Mmp8	*ns*		3.29E^{-07}	**up**	1.44E^{-10}	**up**	*ns*	
Mmp9	*ns*		4.64E^{-04}	**up**	1.13E^{-09}	**up**	*ns*	
Mmp10	*ns*		8.48E^{-04}	**up**	1.99E^{-05}	**up**	*ns*	
Mmp11	*ns*		2.07E^{-02}	**up**	1.85E^{-02}	**up**	*ns*	
Mmp12	4.80E^{-03}	**up**	1.55E^{-11}	**up**	1.04E^{-09}	**up**	*ns*	
Mmp13	*ns*		*ns*		3.68E^{-06}	**up**	7.99E^{-03}	**up**
Mmp14	*ns*		2.82E^{-20}	**up**	2.19E^{-39}	**up**	*ns*	
Mmp19	*ns*		2.13E^{-13}	**up**	1.12E^{-16}	**up**	*ns*	
Mmp24	*ns*		7.16E^{-03}	**down**	*ns*		*ns*	
Ncan	4.80E^{-03}	**down**	8.10E^{-03}	**down**	*ns*		*ns*	
Sdc1	*ns*		4.95E^{-11}	**up**	4.35E^{-15}	**up**	*ns*	
Sdc3	*ns*		9.71E^{-03}	**up**	6.81E^{-13}	**up**	8.54E^{-04}	**up**
Sdc4	1.30E^{-03}	**up**	1.26E^{-23}	**up**	1.68E^{-13}	**up**	*ns*	
Sparcl1	*ns*		8.30E^{-04}	**down**	6.76E^{-08}	**down**	*ns*	
Timp1	*ns*		5.84E^{-25}	**up**	4.99E^{-30}	**up**	*ns*	
Timp2	*ns*		2.15E^{-03}	**up**	7.81E^{-03}	**up**	*ns*	
Timp3	*ns*		1.78E^{-07}	**up**	1.08E^{-05}	**up**	*ns*	
Timp4	*ns*		6.25E^{-03}	**up**	1.37E^{-02}	**up**	*ns*	
Tnc	*ns*		1.46E^{-32}	**up**	8.87E^{-48}	**up**	*ns*	
Vcan	*ns*		3.27E^{-10}	**up**	3.41E^{-17}	**up**	*ns*	
